# The Relationship of Religious Comfort and Struggle with Anxiety and Satisfaction with Life in Roman Catholic Polish Men: The Moderating Effect of Sexual Orientation

**DOI:** 10.1007/s10943-017-0388-y

**Published:** 2017-03-25

**Authors:** Beata Zarzycka, Radosław Rybarski, Jacek Sliwak

**Affiliations:** Department of Social Psychology and Psychology of Religion, The John Paul II Catholic University of Lublin, Al. Racławickie 14, 20-950 Lublin, Poland

**Keywords:** Homosexuality, Religious struggle, Religious comfort, Anxiety, Satisfaction with life

## Abstract

The aim of the research was to analyze the relationships of religious comfort and struggle with state anxiety and satisfaction with life in homosexual and heterosexual samples of men. A hundred and eight men aged between 18 and 43 participated in the research in total, 54 declared themselves as homosexual and 54 as heterosexual. The Religious Comfort and Strain Scale, the State-Trait Anxiety Inventory and the Satisfaction with Life Scale were applied to the research. The results of hierarchical multiple regression analyses revealed that sexual orientation moderated the relationships of religious comfort and struggle with state anxiety and satisfaction with life. The highest state anxiety was observed in homosexual participants with high negative social interactions surrounding religion scores. Negative religious social interactions with fellow congregants and religious leaders, including disapproval and criticism, create anxiety among homosexual people. It seems that homosexual participants are engaged in a trade-off between valued and necessary religious engagement and the harassment and persecution they may be forced to endure in order to access that engagement.

## Introduction

The vast majority of the Polish population consider themselves religious; 97% are Christian, of which 95% are members of the Catholic Church. More than half of Polish Catholics report that they attend religious services (59%) and engage in private acts of devotion such as prayer (73%) at least once a week. The frequency with which Poles engage in public as well as private acts of devotion serves as evidence of the importance of religion in their lives. The significance of Catholic religion in Poland is also evident in the ways in which Poles incorporate their beliefs into everyday life. Religiosity has the greatest influence on family life and relationships, childrearing, events such as birth, marriage and death, friendships and other relationships (Zarzycka 2009).

Although Catholic religion has a powerful influence over many aspects of people’s private lives in Poland, sexuality has always been one of religion’s most important sites of influence. Catholic doctrine informs social norms regarding what constitutes acceptable patterns of sexual intimacy and defines who constitutes an appropriate sexual partner. For homosexual individuals, whose patterns of sexual identities challenge religious norms, religion and religious communities often have been hostile spaces, and their efforts to integrate religion and sexuality are often wrought with conflict (Boczkowski 2003; Halkitis et al. 2009). Furthermore, some members of Catholic communities maintain a posture of intolerance toward homosexual people, which often puts these individuals in a difficult situation (Hamblin and Gross 2014). Religion-related strains in homosexuals may be stronger on the Polish than the Western ground due to a more traditional image of religiosity in Poland.

The results of the research on attitudes of Poles toward minorities suggest that homosexual individuals are subject to the strongest prejudice in Poland (Antosz 2012; (Poleszczuk et al. 2013). According to the Polish Prejudice Survey 2, conducted in 2013 on the representative sample of 965 adult Poles, every third Pole perceives homosexual individuals as a source of threat to traditional values and to the Polish family. This survey was aimed at examining two forms of homophobia: traditional and modern (Morrison and Morrison 2003). Traditional homophobia refers to hostile attitudes toward homosexual individuals, resulting from moral or religious beliefs related to homosexuality. In Poland, the highest level of traditional homophobia was observed among country dwellers, individuals with a lower education level and elderly people. Young, educated city dwellers show these types of prejudice less often. Interestingly enough, the highest percentage of Poles (48%) stated that homosexual individuals should not be allowed to work with children. When it comes to the modern homophobia, a hostile attitude toward homosexual individuals stems from perceiving their political demands as unjustified and from the perceived unwillingness of homosexual individuals to assimilate with the heterosexual majority. Modern types of prejudice toward homosexuals are not related to the education level and are less conditioned by age or the place of living of the respondents. The highest percentage of Poles (53%) stated that homosexual individuals have become too confrontational in their demands for equal treatment. The general level of modern homophobia turned out to be higher in Poland than the level of traditional homophobia (Górska and Mikołajczyk 2014).

Although most Polish homosexual people are raised in families which practice Catholic faith (Boczkowski 2003; Lew-Starowicz and Lew-Starowicz 1999), little have we known about how they experience their religiosity and whether their religious engagement is for them a source of comfort or struggle (cf. Halkitis et al. 2009; Hamblin and Gross 2014). We also know little about the association between religious comfort and struggle and indicators of mental health among Polish homosexual people. This work serves as a corrective to the present gap in knowledge. We examined the relationships of religious comfort and struggle with state anxiety and satisfaction with life among homosexual Poles who are closeted. Next, the moderating effect of sexual orientation on the associations of religious comfort and struggle with state anxiety and satisfaction with life was tested.

### Religious Comfort and Struggle and Mental Health

Involvement in religious activity has been considered a buffer against (for review, see Hill and Pargament 2003; Koenig et al. 2001; Larson and Larson 2003) as well as a contributor to psychological distress (for review, see Ano and Vasconcelles 2005; Exline 2013; Exline and Rose 2013; Pargament 2007; Smith et al. 2003). Religious life may meet the need for relationships, provide tips for coping with adversities and help to shape sense of life (Park 2005). In the last 30 years, psychologists have discovered and described positive functions of religiosity in various areas of social adaptation, mental health and quality of life (see Koenig 1997; Koenig et al. 2001). For example, there have been observed positive correlations between religious involvement and well-being (Koenig 1997), a sense of coherence (Zarzycka and Rydz 2014), life’s satisfaction (Zwingmann 1991), personal adjustment (Koenig et al. 1988; Watson et al. 1994), self-control (Bergin et al. 1987), coping with stress (Pargament 1997). Taking into account the pragmatic advantages granted by religion, it seems obvious to follow the opinion that religion is a source of comfort and a beneficial tool to cope with stress (Exline 2002; Zarzycka 2014).

However, religious life provides not only benefits (Jonas and Fischer 2006; Park 2005), but also strain and internal struggles (Exline et al. 2000). The notion of struggle implies that something in a person’s current belief, practice or experience is causing or perpetuating distress (Exline 2013). Religion may be the source of stress if it focuses people’s attention on their sinfulness and the prospect of God’s punishment (Exline 2002; Pargament et al. 2005; Virkler 1999). Normative rules in religion happen to be a challenge and oblige people to take actions that evoke discomfort (Exline 2002). People are not unanimous in basic issues regarding the religious doctrine or they feel disappointed with the religious institution (Krause et al. 2000). Surveys using nonclinical samples have shown consistent relationships between spiritual struggle and poor adjustment (e.g., Ano and Vasconcelles 2005), depression (e.g., Smith et al. 2003) and anxiety (e.g., McConnell et al. 2006). Studies involving medical conditions have showed that religious struggle is associated with greater emotional distress for blood and marrow transplant patients (King et al. 2013), diabetes (Fitchett et al. 2004), patients facing cardiovascular problems (e.g., Magyar-Russell et al. 2014), cancer (e.g., Edmondson et al. 2008), asthma (e.g., Benore et al. 2008) and congestive heart failure (e.g., Park et al. 2009).

### Conflict Between Religion and Sexual Orientation

Most denominations within the Christian traditions, particularly the Catholic Church, have been unaccepting of homosexuality. For Christians, the Bible states that: “If a man lies with a male as with a woman, both of them have committed an abomination; they shall surely be put to death; their blood is upon them” (Lev, 20:13, Millennium Bible Version). Official Catholic teachings, although lately careful to distinguish between homosexual orientation and homosexual behavior, continue to strongly oppose homosexual sexuality (Walker and Longmire-Avital 2013). According to this view, because such relationships are deprived of any procreative possibility and are not consolidated by the bond of marriage, homosexual acts are considered to be in possession of characteristics that render the homosexual inclination *objectively disordered* and view same-sex sexual activities as sinful or unnatural (Congregation for the Doctrine of the Faith 1986; cf. Dèttore et al. 2014; Kralovec et al. 2014). As a result, many homosexual people report that their religion is more of a struggle than a comfort (Hamblin and Gross 2014; Henrickson et al. 2007). The struggles are potentially very difficult, especially if the person belongs to one of the mainstream Christian denominations or comes from a family that upholds the teachings of these denominations (Rodriguez and Ouellette 2000; Sowe et al. 2014; Subhi and Geelan 2012). Sources of conflict included denominational teachings, scriptural passages and congregational prejudice (Schuck and Liddle 2001). The exposure to religious environments that do not affirm homosexuality may lead to internalized homophobia, depressive symptoms, less psychological well-being and even suicidal ideation (Barnes and Meyer 2012).

### Religion and Psychological Health Among Homosexual People

The previous research results have suggested that religion may play a role in various aspects of well-being among homosexual people (Hamblin and Gross 2014). Tan (2005) observed that gay and lesbian respondents who identified as having a formal religion scored higher on the measure of religious well-being (relation to God). Religious well-being scores did not significantly predict measured aspect of adjustment (self-esteem, feelings of alienation or depression); however, religious well-being and overall life satisfaction were highly correlated. The author suggested that religion is not an important component of the lives of gay men and lesbians, but that the existential component of spirituality is important for adjustment. The results indicated that existential well-being was a significant predictor of higher self-esteem, lower internalized homophobia and less alienation. Yakushko (2005) recruited a small sample of gay, lesbian and bisexual participants at a conference supporting the movement toward full acceptance of homosexuality in Christian denomination. The results suggested that attendance of a conservative (rejective–punitive) church at some point significantly related to lower self-esteem and higher stress over sexual orientation. Lease et al. (2005) observed that affirming faith experience is beneficial to the psychological well-being of gay men and lesbians although affirming faith experience was not directly related to psychological well-being, but they did have an indirect effect through a combined negative relationship to internalized homonegativity and positive relationship with spirituality. Hamblin and Gross (2014) observed that among gay and lesbian participants who rated their church as rejecting of homosexuality, more frequent attendance of religious services was related to increased symptoms of anxiety; there was no relation between frequency of attendance and anxiety for participants attending accepting communities. They suggested that participation in rejecting religious communities might adversely affect the psychological health of gay and lesbians, while participation in accepting faith communities may offer a source of social support.

### Research Problem

The subject of this research is the analysis of relationships between religious comfort and struggle with state anxiety and satisfaction with life in homosexual men. The research results suggest that religion can be both a source of consolation (e.g., Lease et al. 2005; Tan 2005) and distress (e.g., Hamblin and Gross 2014) for homosexual people. However, this information is based on single indicators of religious commitment, e.g., religious well-being (e.g., Tan 2005), spirituality (e.g., Lease et al. 2005) and church attendance (e.g., Hamblin and Gross 2014). Furthermore, the majority of studies have been carried out in gay or lesbian samples who identify themselves as gay. The research on homosexual people who do not accept their sexual orientation and hide it is scarce.

The present research included two religious dimensions—religious comfort and struggle. Religious comfort is an indicator of benefits derived from faith and relation to God (Jonas and Fischer 2006). Religious struggle includes three categories: preoccupation with one’s own guilt and feeling unforgiven by God, negative emotions toward God and negative social interactions surrounding religion. We compared how religious comfort and struggle correlate with satisfaction with life and anxiety in homo- and heterosexual men. We focused on state anxiety over trait anxiety because of an interest in quantifying the effect of the religion measures on the respondents. Next, we examined whether the correlations that exist between religious comfort/struggle and state anxiety and satisfaction with life are moderated by sexual orientation.

The authors formulated two hypotheses. The first one refers to the knowledge about the supportive function of religious comfort and the weakening function of religious struggle within mental health. The assumption is that religious comfort correlates positively with satisfaction with life and negatively with state anxiety—in both homo- and heterosexual group. When it comes to religious struggle, reverse correlations were expected—negative with satisfaction with life and positive with state anxiety. The second hypothesis assumes that the relationship of religious comfort and struggle with anxiety and satisfaction with life is moderated by sexual orientation. First, the expectation was that homosexual individuals, as they experience current conflict, are more willing to activate their religious resources, i.e., they derive more support from religion than their heterosexual counterparts. Consequently, religious comfort will be a stronger predictor of satisfaction with life in a homosexual group than in the heterosexual one. Second, it was expected that negative social interactions surrounding religion will be a stronger state anxiety predictor in homosexual individuals than in heterosexual ones. Reports on social situation of homosexual individuals in Poland show that the majority of the respondents are discriminated because of their sexual orientation (Abramowicz 2007; Krzemiński 2009). Experienced discriminating attitudes may result in rise in state anxiety. The analysis of the moderation effect of sexual orientation on the relationships between fear–guilt, negative emotions toward God and satisfaction with life and state anxiety is of exploratory nature.

## Methods

### Sample

We used a screening question “How do you describe yourself?” with four multiple choice answers (heterosexual, bisexual, homosexual and not sure) before the survey started. We applied snowball sampling recruitment. We recruited initially 41 students from two Polish universities, and 9 individuals who were not students. The rest of them were recruited through recommendations. Of 155 people contacted, 123 (79%) returned the questionnaire with *n* = 54 appropriate for analysis. Exclusion included being bisexual, incomplete answers, not sure answers or responses past the deadline. All respondents were closeted and did not identify themselves as gay. The mean age of the homosexual sample was 26.40 years (SD = 5.59). Their highest education level completed was high school or graduate school. The majority of them were single, living in urban environment. All of them declared Roman Catholic affiliation in which they were brought up (see Table 1).

**Table 1:** Demographic characteristic of participants (*n* = 108)

Characteristic	Homosexual		Heterosexual	
	*n*	%	*n*	%
Highest education level completed				
High school	33	61.11	32	59.26
Graduate school	21	38.89	22	40.74
Place of living				
Village	18	33.33	17	31.48
City or town below 200,000	15	27.78	18	33.33
City above 200,000	21	38.89	19	35.19
Marital status				
Single	48	88.89	31	57.41
Married	4	7.41	21	38.89
Divorced	2	3.70	2	3.70

A heterosexual control group was matched to the homosexual sample with respect to sex, age, degree of education and place of living. Participants were recruited from two Polish universities. Of 189 people contacted, 156 (82%) returned the questionnaire with *n* = 132 appropriate for analysis. After exclusion of those participants who were not comparable to homosexual participants, *n* = 54 remained for final analysis. The mean age of the heterosexual sample was 27.73 (SD = 5.82). Their highest education level completed was high school or graduate school; they were single or married, living mostly in urban surroundings. All of them declared Roman Catholic affiliation in which they were brought up (see Table 1).

### Measures

Participants responded to paper-and-pencil measures of religious comfort and struggle, state anxiety and satisfaction with life. The religion measure preceded the anxiety and satisfaction with life measures.

Religious comfort and struggle was assessed using Religious Comfort and Strain Scale (RCSS) (Exline et al. 2000; cf. Zarzycka 2014). It is a set of 24 face-valid items designed to assess the degree to which participants are experiencing feelings of comfort and three types of struggle associated with religion (Exline 2013; Exline et al. 2000). Participants are asked the following question: “To what extent are you currently having each of these experiences?” They focus on their general perceptions, feelings or attitudes rather than their coping responses to a specific stressor. Items are rated on an 11-point Likert scale (0 = *not at all*; 11 = *extremely*). Polish version of the RCSS consists of four subscales (Zarzycka 2014):

- *Religious comfort* (*α* = .96) sense of trust toward God, perceiving God as almighty, supportive and taking care of people, perceiving faith as a source of strength, peace, harmony, sense of meaning and purpose in life.
- *Negative emotions toward God* (*α* = .86) negative feelings toward God; perceiving God as unfair, untrustworthy, cruel and abandoning people;
- *Fear*–*guilt* (*α* = .74) preoccupation with one’s own sin, guilt; feeling unforgiven by God.
- *Negative social interactions surrounding religion* (*α* = .56) negative emotions and relationships with fellow congregants;

State anxiety was measured using the State-Trait Personality Inventory (STAI) (Spielberger et al. 1983). STAI indicates the intensity of feelings of anxiety. It distinguishes between state anxiety (a temporary condition experienced in specific situations) and trait anxiety (a general tendency to perceive situations as threatening) (Spielberger et al. 1983). It consists of 40 items, 20 items for assessing trait anxiety and 20 for state anxiety. Both scales were intended to form unidimensional measures. For the state items, respondents are asked to indicate “How you feel right now, that is, at this moment” (e.g., I am tense; I am worried; I feel calm; I feel secure). Responses indicate intensity of feeling on a 1–4 scale, from 1 = *not at all* to 4 = *very much so*. For the trait items, the question concerns “how you generally feel” (e.g., I worry too much over something that really doesn’t matter; I am content; I am a steady person). The response scale indicates frequency from 1 = *almost never* to 4 = *almost always*. In the research, we focused on state anxiety over trait anxiety because we intended to measure the effect of the respondents’ religious comfort and struggle on their feeling of anxiety. Internal consistency coefficients for the State Anxiety Scale have ranged from .87 to .91. Test–retest reliability coefficients have ranged from .65 to .75 over a 2-month interval (Spielberger et al. 1983). In this sample, the internal consistency coefficients for the state anxiety scales were .69.

Satisfaction with life was assessed using the Satisfaction with Life Scale (SWLS) by Diener et al. (1985). The SWLS is a short 5-item instrument designed to measure global cognitive judgments of satisfaction with one’s life. Respondents indicates the extent to which they agreed with each item on a seven-point Likert scale ranging from 1 = *strongly agree* to 7 = *strongly disagree*. Several factor-analytic studies have supported a unidimensional structure of the SWLS (e.g., Arrindell et al. 1991; Neto 1993), as it was conceptualized by Diener et al. (1985). The psychometric properties of the SWLS have been tested in numerous studies. Internal consistency has been shown to generally exceed values around .80 (Diener et al. 1985). The psychometric properties of Polish version of the SWLS were also satisfactory. Internal consistency coefficient was .72. Test–retest reliability coefficients were .83 over a 2-week interval and .84 over a 1-month interval (Juczyński 2001). In this sample, the internal consistency coefficients for SWLS were .89.

## Results

The analysis of relations of religious comfort and struggle to satisfaction with life and state anxiety was conducted by means of correlation methods. First, the authors applied the *t* test to establish differences between homo- and heterosexual groups in terms of the variables tested. Next, the correlation matrix (*r*-Pearson) between the variables was calculated. Hierarchical regression analysis with the interaction component was the last stage. It was aimed at checking whether sexual orientation moderates relations of religious comfort and struggle to satisfaction with life and state anxiety.

### Descriptive Data

Participants showed a significant difference on their reported satisfaction with life and religious comfort scores based on their sexual orientation. Heterosexual men reported higher level of satisfaction with life [*M* = 19.20, SD = 5.46, *t*(94) = 19.20, *p* < .01] than did homosexual men (*M* = 15.73, SD = 5.22). Homosexual men reported higher level of religiosity [*M* = 4.89, SD = .90, *t*(94) = 3.47; *p* < .001] and higher religious comfort [*M* = 8.11, SD = 1.45, *t*(94) = 8.11, *p* < .001] than did heterosexual men (religiosity: *M* = 4.19, SD = 1.11, religious comfort: *M* = 6.34, SD = 2.82). The groups did not differ on reported state anxiety, negative emotions toward God, fear–guilt or negative social interactions surrounding religion.

### Relationships of Religious Comfort and Struggle with State Anxiety and Satisfaction with Life

In the homosexual group, satisfaction with life correlated positively with religious comfort and negatively with negative social interactions surrounding religion, while state anxiety correlated positively with all measured types of religious struggle. In the heterosexual group, state anxiety correlated negatively with religious comfort and positively with both fear–guilt and negative emotions toward God, while state anxiety correlated positively with all measured types of religious struggle. Satisfaction with life correlated negatively with three types of religious struggle (see Table 2).

**Table 2:** Correlation of study variables

Variable	1	2	3	4	5	6
1. State anxiety	–	−.56***	−.16**	.38**	.50***	.23
2. Satisfaction with life	−.39**	–	.16	−.26*	−.37**	−.29*
3. Religious comfort	−.23	.29*	–	−.30*	.13	−.17
4. Negative emotions toward God	.33*	−.12	−.65***	–	.56**	.43**
5. Fear–guilt	.37*	−.17	−.45**	.73***	–	.28*
6. Negative social interactions	.49***	−.42**	−.22	.40**	.51***	–

Correlations observed in homosexual men groups are presented under the diagonal and in heterosexual men groups—over the diagonal

* *p* < .05; ** *p* < .01; *** *p* < .001

A two-step hierarchical linear regression was conducted to determine whether sexual orientation, religious comfort and struggle, and their interactions significantly predicted state anxiety and satisfaction with life. The independent variables were centered; in the case of the religious comfort and struggle, the method based on the standardization variable results was chosen.

### Religious Comfort and Struggle and State Anxiety

Fear–guilt was the only variable contributing significantly to the model in the first step. Fear–guilt predicted 26% of the variance in participant state anxiety (*p* < .01), while sexual orientation, religious comfort, negative emotions toward God and negative social interactions surrounding religion were not significant: regardless of reported sexual orientation, religious comfort, negative emotions toward God and negative social interactions surrounding religion, those with increases in fear–guilt were more anxious. The hierarchical linear regression with interactive component showed that sexual orientation, religious comfort and negative emotions toward God did not contribute significantly to the model in the second step. However, the interaction terms of sexual orientation and fear–guilt as well as sexual orientation and negative social interactions surrounding religion contributed 5% (*R* ^2^ change = .05, *p* < .05) to the model and were significant predictors of state anxiety. The model with the interactive component was well suited to the data *F*(7, 88) = 5.85; *p* < .001 (see Table 3, Step 2).

**Table 3:** Hierarchical regression analysis summary for sexual orientation, religious comfort and strain variables, and their interactions predicting state anxiety (*n* = 108)

Step and variable	*β*	95% CI	*t*	*p*
Intercept		(37.45, 41.38)		<.001
Step 1				
Sexual orientation	.12	(−.83, 3.46)	1.22	.227
Religious comfort	−.13	(−3.82, .98)	−1.18	.242
Negative emotions toward God	.02	(−2.80, 3.25)	.15	.883
Fear–guilt	.37	(−1.31, 6.75)	2.95	<.01
Negative social interactions	.16	(−.58, 4.07)	1.49	.139
		*R* = .52		
		*R* ^2^ = .26		
Step 2				
Sexual orientation	.15	(−.44, 3.78)	1.57	.119
Religious comfort	−.17	(−4.28, .51)	−1.56	.122
Negative emotions toward God	.04	(−2.50, 3.41)	.31	.758
Fear–guilt	.30	(.50, 5.96)	2.35	<.05
Negative social interactions	.24	(.23, 4.99)	2.18	<.05
Orientation * fear–guilt	−.23	(−4.77, −.28)	−2.24	<.05
Orientation * interaction	.23	(.27, 4.85)	2.22	<.05
		*R* = .56		
		*R* ^2^ = .32		

*CI* confidence interval

**Fig. 1:**
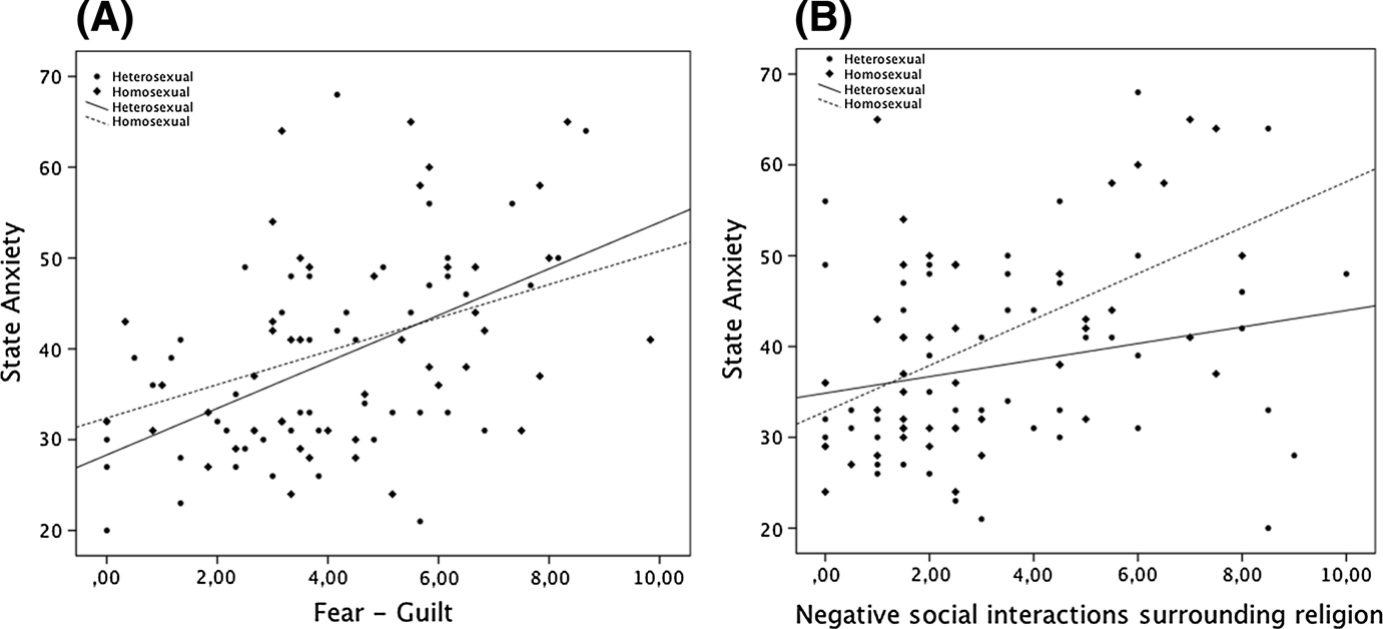
Level of state anxiety as a function of sexual orientation and **a** fear–guilt or **b** negative social interactions surrounding religion. Computed slopes of regression line for level of **a** fear–guilt and state anxiety and **b** negative social interactions surrounding religion and state anxiety are presented

A single slope analysis was conducted to examine the significance of the interactions terms. Participants who reported heterosexual orientation and lower fear–guilt scores reported the lowest state anxiety, while those reporting heterosexual orientations and higher fear–guilt scores reported the highest state anxiety (see Fig. 1a). Participants who reported homosexual orientation and lower negative social interactions surrounding religion scores reported the lowest state anxiety, while those reporting homosexual orientation and higher negative social interactions surrounding religion scores reported the highest state anxiety (see Fig. 1b).

To examine the performance of the model for homosexual and heterosexual groups, the model was run again twice, once for participants reporting homosexual orientation and again for those reporting heterosexual orientation. Religious comfort or negative emotions toward God did account for a significant portion of the variance in predicting state anxiety neither in the homosexual nor in the heterosexual group. However, for participants reporting homosexual orientation, negative social interactions surrounding religion contributed to their state anxiety, while for those reporting heterosexual orientation fear–guilt accounted for a significant portion of the variance in predicting state anxiety (see Table 4).

**Table 4:** Regression analysis summary for religious comfort and strain variables predicting state anxiety in homo- and heterosexual groups

Variables	Homosexual group				Heterosexual group			
	*β*	95% CI	*t*	*p*	*β*	95% CI	*t*	*p*
Intercept		(12.28, 63.92)	2.98	<.01		(24.37, 41.05)	7.89	<.001
Religious comfort	−.09	(3.33, 1.95)	−.53	.600	−.21	(−1.82, .24)	−1.54	.131
Negative emotions toward God	.09	(−2.02, 3.01)	.40	.691	.01	(−1.64, 1.76)	.07	.946
Fear–guilt	.03	(−2.01, 2.33)	.15	.882	.55	(1.13, 4.16)	3.52	<.001
Negative social interactions	.41	(.36, 3.68)	2.47	<.05	.01	(−1.04, 1.15)	.10	.920
		*R* = .53				*R* = .58		
		*R* ^2^ = .28				*R* ^2^ = .33		

*CI* confidence interval

### Religious Comfort and Struggle and Satisfaction with Life

Sexual orientation, fear–guilt and negative social interactions surrounding religion contributed significantly to the model in the first step and accounted for in total 28% of the variance in satisfaction with life (*p* < .001). Religious comfort and negative emotions toward God were not significant: regardless of religious comfort and negative emotions toward God, those with reported heterosexual orientation, decreases in fear–guilt and negative social interactions surrounding religion were more satisfied with life (see Table 5). The hierarchical linear regression with interactive component (Step 2) showed that the interaction term of sexual orientation and fear–guilt contributed 6% to the model and was significant predictor of satisfaction with life (*R* ^2^ change = .56, *p* < .05). The model with interactive component was well suited to the data *F*(7, 88) = 6.34; *p* < .001 (see Table 5).

**Table 5:** Hierarchical regression analysis summary for sexual orientation, religious comfort and strain variables and their interactions predicting satisfaction with life (*n* = 108)

Step and variable	*β*	95% CI	*t*	*p*
Intercept		(16.43, 18.41)		<.001
Step 1				
Sexual orientation	−.36	(−3.10, −.92)	−3.68	<.001
Religious comfort	.17	(−.30, 2.15)	1.54	.127
Negative emotions toward God	.20	(−.37, 2.69)	1.51	.134
Fear–guilt	−.29	(−2.96, −.21)	−2.30	<.05
Negative social interactions	−.31	(−2.93, −.60)	−2.96	<.01
		*R* = .53		
		*R* ^2^ = .28		
Step 2				
Sexual orientation	−.39	(−3.27, −1.14)	−4.11	<.001
Religious comfort	.22	(.06, 2.47)	2.08	<.05
Negative emotions toward God	.19	(−.40, 2.60)	1.45	.151
Fear–guilt	−.23	(−2.63, .12)	−1.82	.073
Negative social interactions	−.37	(−3.29, .89)	−3.45	<.001
Orientation * fear–guilt	.27	(.38, 2,64)	2.66	<.01
Orientation * interaction	−.17	(−2,09, .21)	−1.62	.110
		*R* = .58		
		*R* ^2^ = .34		

*CI* confidence interval

**Fig. 2:**
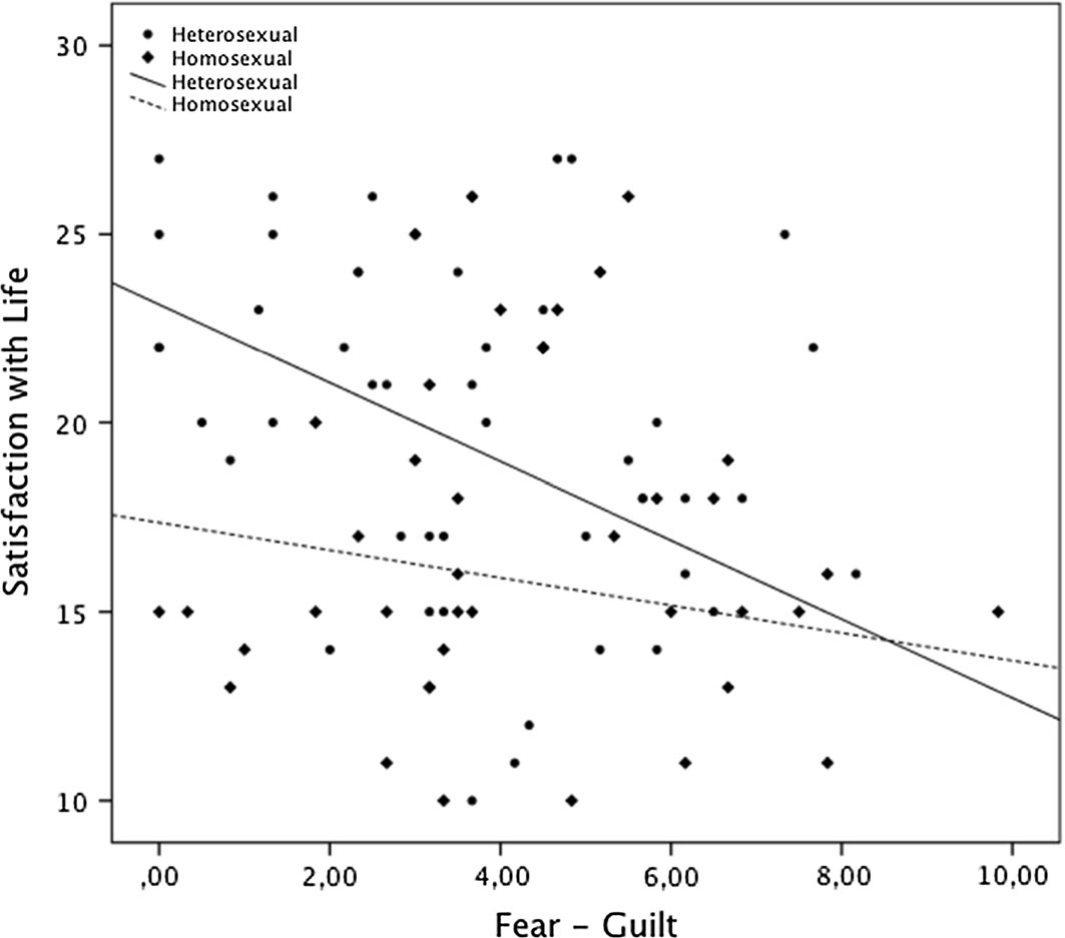
Level of satisfaction with life as a function of sexual orientation and fear–guilt. Computed slopes of regression line for level of fear–guilt and satisfaction with life are presented

A single slope analysis was conducted to examine the significance of the interactions terms. Participants who reported heterosexual orientation and lower fear–guilt scores reported the highest satisfaction with life, while those reporting higher fear–guilt scores and heterosexual orientation reported the lowest satisfaction with life (see Fig. 2).

The analysis of variable relations: religious comfort, negative emotions toward God, fear–guilt and negative social interactions surrounding religion with satisfaction with life in the groups separated according to sexual orientation, has shown that the relation between fear–guilt and satisfaction with life is stronger and negative in heterosexual men, whereas in homosexual men this relation is insignificant. In addition, a positive correlation of satisfaction with life with religious comfort and a negative one with z negative social interactions surrounding religion was observed in homosexual men (see Table 6).

**Table 6:** Regression analysis summary for religious comfort and strain variables predicting satisfaction with life in homo- and heterosexual groups

Variables	Homosexual group				Heterosexual group			
	*β*	95% CI	*t*	*p*	*β*	95% CI	*t*	*p*
Intercept		(−5.51, 18.16)	1.08	.286		(17.43, 26.42)	9.82	<.001
Religious comfort	.35	(.06, 2.48)	2.12	<.05	.19	(−.19, .93)	1.34	.187
Negative emotions toward God	.27	(−.46, 1.84)	1.21	.234	.18	(−.46, 1.37)	1.00	.323
Fear–guilt	.08	(−.79, 1.18)	.40	.694	−.48	(−1.98, −.36)	−2.90	<.01
Negative social interactions	−.49	(−1.87, −.36)	−2.97	<.01	−.24	(−1.07, .11)	−1.64	.108
		*R* = .55				*R* = .51		
		*R* ^2^ = .30				*R* ^2^ = .26		

*CI* confidence interval

## Discussion

There is ample data confirming positive functions of religiosity with relations to health in a general population (for review, see Hill and Pargament 2003; Koenig et al. 2001; Larson and Larson 2003). Psychologists have described effects of religious comfort and struggle on different aspects of social adaptation, well-being and quality of life both in general population and in clinical samples (see Koenig 1997; Koenig et al. 2001). However, we still know very little about how religious comfort and struggle work in homosexual people (Hamblin and Gross 2014; Hancock 2000; Yakushko 2005). It has been suggested that repeated exposure to negative messages within religious bodies may have destructive consequences to the mental health of homosexual people. On the contrary, positive and affirming religious community may be a positive resolution to conflict between religion and sexual orientation (c.f. Hamblin and Gross 2014; Rodriguez and Ouellette 2000).

The research presented in this paper was aimed at analyzing relationships of religious comfort and struggle with state anxiety and satisfaction with life in homosexually identified Roman Catholic Polish men. The results observed let us suggest that religiosity and homosexuality do not have to be reciprocally excluding (cf. Walker and Longmire-Avital 2013). Religious homosexual men derived more support from religion than their heterosexual counterparts. Religious comfort was also a significant predictor of satisfaction with life in the homosexual group. Tan (2005) observed similar results; in his research, gay and lesbian respondents who were identified as having a formal religion scored higher on the measure of relation to God. However, relation to God was not the only predictor of personal adaptation, but it revealed strong relations with overall satisfaction with life. The obtained results may be interpreted by reference to knowledge about the buffering function of religiosity in a difficult situation. Religious homosexual individuals find themselves in a situation of current conflict between their religious faith and sexual orientation (Schuck and Liddle 2001). In addition, these individuals often come across manifests of lack of acceptance and even discrimination in some family, school and workplace environments (Sowe et al. 2014). Discovering religion as a personal resource and the ability to derive benefits from faith may increase sense of security and satisfaction with life in this group. Moreover, satisfaction with life in homosexual individuals is conditioned by the lack of strain in relations to fellow congregants. The results of studies confirm that religious faith plays a supportive role not only directly, through a system of religious beliefs and ability to assign meanings (cf. Park et al. 2013), but also indirectly, including the fact of being source of social support from a group of believers (Park and Slattery 2013). To the extent that the religious community is rejecting and discriminatory, involvement in structured religious activities may reduce satisfaction with life in homosexual men.

Homosexual individuals did not differ significantly from the heterosexual control group in terms of intensity of religious struggle. Moreover, negative emotions toward God, fear–guilt and negative social interactions surrounding religion correlated positively with state anxiety. These results confirmed a negative effect of religious struggle on many indicators of emotional distress, observed both in a normal population and in medical samples (Exline and Rose 2013). For example, McConnell et al. (2006) surveyed a random sample of 1629 respondents and observed positive links between spiritual struggle and all of the study’s distress measures: depression, paranoid ideation, somatization and several indicators of anxiety. Another recent study using a large sample (Ellison and Lee 2010) also showed connections between distress and intrapsychic, divine and interpersonal struggle. Studies of nonclinical samples of Jews, Muslims, Christians and undergraduates have yielded comparable patterns (Exline and Rose 2013).

The analysis of hierarchical regression confirmed the moderating effect of sexual orientation on the relationship of state anxiety with strain in relations to fellow congregants and religious preoccupation with own guilt. In homosexual men, strain with religious leaders and fellow congregants increase state anxiety significantly, whereas this relation is not significant in heterosexual men. This means that what homosexual individuals are afraid of in religion are judgments and assessments formulated by those who represent religious institutions.

Social support is one of the most obvious benefits to those involved in organized religion. Furthermore, the social support people gain through their religious involvement may be qualitatively different than secular social support. Religious social support reinforces and is reinforced by a collective framework of ultimate meaning, belongingness and cohesion in ways that may not be matched by secular groups (Park and Slattery 2013). Negative religious social interactions with fellow congregants and religious leaders, including disapproval and criticism, create distress and state anxiety. It seems that homosexual participants are engaged in a trade-off between valued and necessary religious engagement and the harassment and persecution they may be forced to endure in order to access that engagement.

State anxiety in homosexual men is increased significantly by preoccupation with one’s own guilt and feeling unforgiven by God. Previous studies have confirmed that religion is related to the experience of guilt and that religious individuals experience more feelings of guilt (e.g., Luyten et al. 1998). The majority of religious systems, in particular the Catholic Church, teach that that man got distanced from God through sin and needs forgiveness. When misunderstood, the command to strive for religious perfectness also increases guilty feeling by excessive preoccupation with personal weaknesses (Exline et al. 2000). Religion intensifies preoccupation with guilt and fear of God in the heterosexual group. So heterosexual men struggle because of their preoccupation with their own sin and the feeling of inadequacy in front of God—in contrast to homosexual men who got significantly more support from their own faith and relation to God but were also afraid of assessments by believers. Interesting is the fact that although preoccupation with own guilt belongs to the area of negative feelings, it has positive implications for personal and social functioning—it facilitates pro-social behaviors of an individual and empathy (Luyten et al. 1998).

The research conducted can also be applied. The results suggest that clinicians who work with homosexual individuals should take into account religious context and present religious involvement of their patients (cf. Barnes and Meyer 2012). Exposure to negative social interactions with religious people and fellow congregants as well as looking for strategies for managing stress should be a subject of this conversation. Reduction and leveling of prejudice toward homosexual individuals seems to be a significant condition of attaining good psychological health and stronger sense of well-being (Eliason and Schope 2007).

There are several limitations of this study that deserve consideration. Of utmost importance is the exclusive quantitative assessment of complex questions. A qualitative element for participants to explain their feelings and thoughts more fully and exactly would be an interesting addition in efforts to further understand this complex and developing issue. Another methodological limitation is the sampling of participants. With only 54 homosexual participants, the sample size was a limitation of this study. With so much public scrutiny surrounding the sensitive topic in Poland, it is difficult to find and recruit homosexual people to participate in such a study. Nevertheless, recruitment and data collection is continuing for further research with a larger sample. The participant pool for the present study consisted of individuals who are closeted. Further research will need to include homosexual individuals involved in gay organizations, and therefore already at least somewhat open and affirming in their homosexuality in order to have compare those who have not accepted their homosexual orientation with those who have (cf. Matty 2014). In summary, further research should investigate a larger, more diverse sample of religious homosexual individuals. This sample should include individuals who integrate by denying faith or denying their homosexuality, along with those who accept and integrate both. Future studies should examine how individual’s history contributes to the relationship between religious faith and mental health indices.
